# Population variation and differences in serum leptin independent of adiposity: a comparison of Ache Amerindian men of Paraguay and lean American male distance runners

**DOI:** 10.1186/1743-7075-3-34

**Published:** 2006-08-30

**Authors:** Richard G Bribiescas, Matthew S Hickey

**Affiliations:** 1 Reproductive Ecology Laboratory, Department of Anthropology, Yale University, New Haven, CT 06520–8277, USA; 2 Department of Health and Exercise Science, 220 Moby-B Complex, Colorado State University, Fort Collins, CO 80523–1582, USA

## Abstract

**Background:**

Serum leptin variation is commonly associated with fat percentage (%), body mass index (BMI), and activity. In this investigation, we report population differences in mean leptin levels in healthy men as well as associations with fat % and BMI that are independent of these factors and reflect likely variation resulting from chronic environmental conditions.

**Methods:**

Serum leptin levels, fat %, and BMI were compared between lean American distance runners and healthy Ache Native Americans of Paraguay. Mean levels were compared as were the regressions between fat %, BMI, and leptin. Comparisons were performed between male American distance runners (n = 13, mean age 32.2 ± 9.2 SD) and highly active male New World indigenous population (Ache of Paraguay, n = 20, mean age 32.8 ± 9.2) in order to determine whether significant population variation in leptin is evident in physically active populations living under different ecological circumstances independent of adiposity and BMI.

**Results:**

While the Ache were hypothesized to exhibit higher leptin due to significantly greater adiposity (fat %, Ache 17.9 ± 1.8 SD; runners 9.7 ± 3.2, p < 0.0001), leptin levels were nonetheless significantly higher in American runners (Ache 1.13 ng/ml ± 0.38 SD; runners 2.19 ± 1.15; p < 0.007). Significant differences in the association between leptin and fat % was also evident between Ache and runner men. Although fat % was significantly related with leptin in runners (r = 0.90, p < 0.0001) fat % was negatively related in Ache men (r = -0.50, p < 0.03).

**Conclusion:**

These results illustrate that chronic ecological conditions in addition to activity are likely factors that contribute to population variation in leptin levels and physiology. Population variation independent of adiposity should be considered to be an important source of variation, especially in light of ethnic and population differences in the incidence and etiology of obesity, diabetes, and other metabolic conditions.

## Background

Leptin is a polypeptide hormone that is secreted primarily by adipose tissue and acts as a lipostat to the hypothalamus affecting numerous aspects of physiology including metabolism, immune function, and reproduction [1]. While leptin is often highly correlated with fat percentage, other sources of variation are important. Diet [2], activity [3], genetics [4], and infectious status [5], all contribute to leptin variation to some extent independent of adiposity. Other tissues besides fat also contribute to leptin levels although their relative contribution is minor [6].

**Figure 1:**
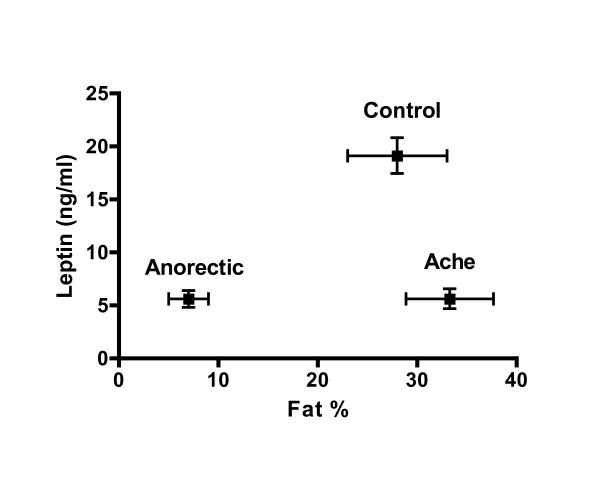
**Mean leptin comparisons between Ache women and American anorectics and controls**. Mean comparisons of leptin and fat percentage between Ache women (leptin 5.6 ± 3.2 ng/ml; fat % 33.3 ± 4.4 SD), American Anorectics (5.6 ± 3.7; 7.0 ± 2.0), and American controls (19.1 ± 8.1; 28.0 ± 5.0) (Ache leptin vs. Anorectic, p < 0.98; Control, p < 0.0001; Ache fat % vs. Anorectic, p < 0.0001; Control, p < 0.004) [11].

Although adiposity is a major source of variation in leptin levels, significant population variation is evident with leptin being significantly lower in non-industrialized populations compared to more developed communities [7-9]. The source of this variation remains to be fully described but chronic environmental and nutritional conditions have been suggested [10]. Among Ache Amerindian women of Paraguay, such marked variation has been reported. Curiously, Ache women exhibit leptin levels that are not significantly different from American anorectic women despite significantly greater adiposity (Figure 1) [11]. Comparisons of leptin among Ache men are less well described.

To determine whether differences in the association between leptin and adiposity extend also to Ache men, this investigation compares Ache male leptin with a group of comparable adiposity in a developed society, American runners. The significance of this comparison is gain insights into ethnic variation in leptin physiology and to provide a greater understanding of the etiology of metabolic syndrome among Native American groups [12,13]. Both groups are lean and exert significant physical activity. While the Ache do not engage in habitual exercise, their activity levels and aerobic profiles reflect significant daily physical exertion resulting from their daily regimen of manual farming and foraging for food products in the surrounding forest [14]. This comparison allows the assessment of interpopulation variation in leptin associations with adiposity within a non-pathological context. It was therefore hypothesized that according to our current understanding of leptin physiology, 1) leptin should be higher in the group exhibiting greater adiposity; 2) leptin should be positively associated with adiposity in each group.

## Methods

Leptin and anthropometric data was collected from the Ache by the author and compared with pre-exercise control measures from runners reported in Hickey et al. [15]. Specific anthropometric and hormone assessment methods are available from their respective references [9]. Although leptin assay methods and kits are highly correlated [16], these data sources were chosen since each utilized similar leptin iodine 125 based radioimmunoassays, thereby reducing interlaboratory variability. Unpaired 2 tailed t-tests of fat%, BMI, age, and leptin were conducted. Welch's correction was employed when variances were significantly different. Standard linear regression was used to determine associations between anthropometric measures and leptin. Comparison of regression slopes was conducted using the methods of Zar, 1999 [17]. Results were calculated using Prism 4.0 for Macintosh (GraphPad Software, San Diego, CA). Alpha was set at 0.05. These protocols were approved by the human subjects protection committees of Yale and Colorado State Universities.

## Results

**Figure 2:**
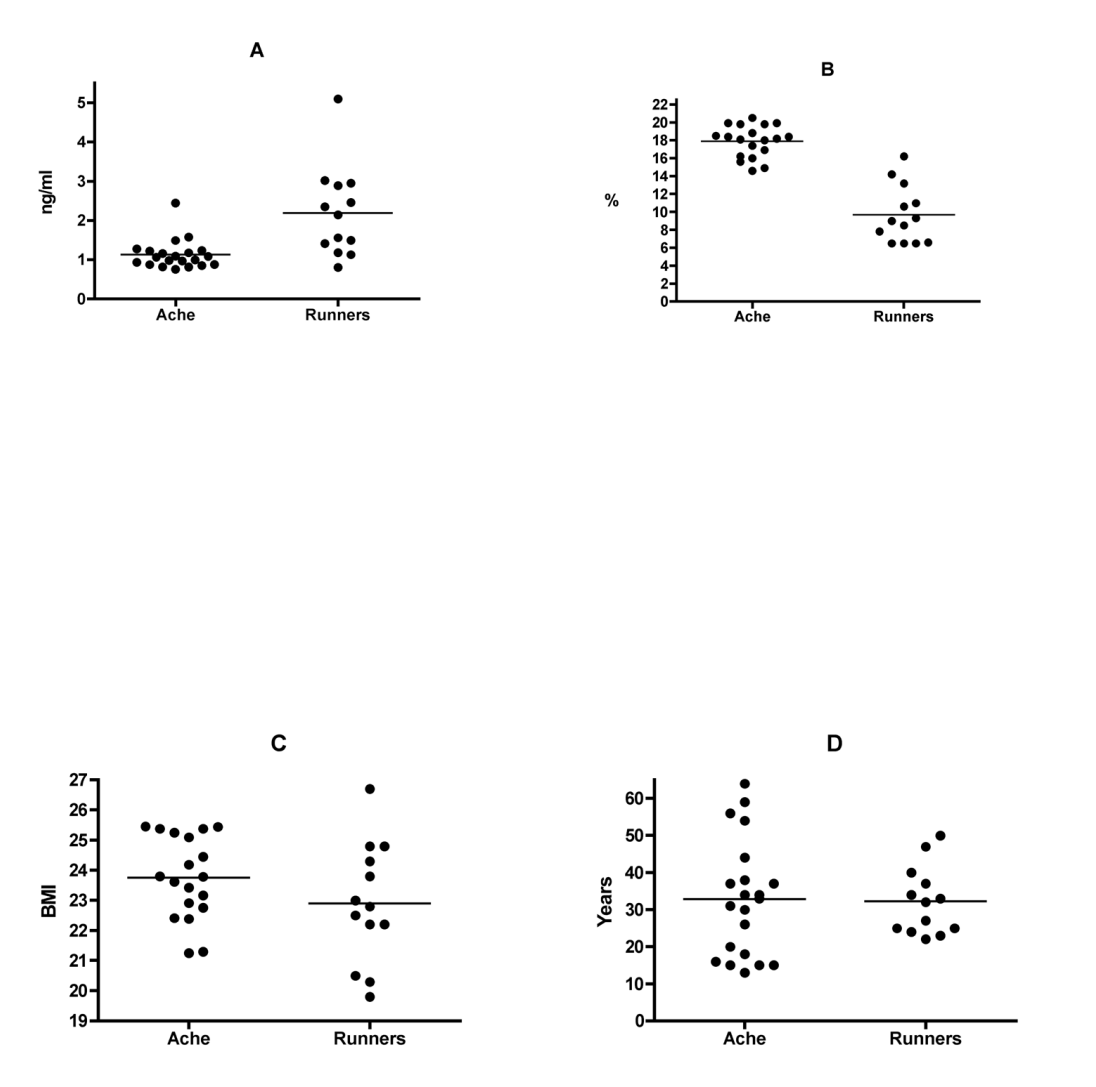
**Mean leptin comparisons between Ache men and American male runners**. Mean comparisons between Ache men and runners (a) Leptin (p < 0.007), (b) Fat % (p < 0.0001), (c) BMI (p < 0.16), and (d) age (p < 0.89).

**Figure 3:**
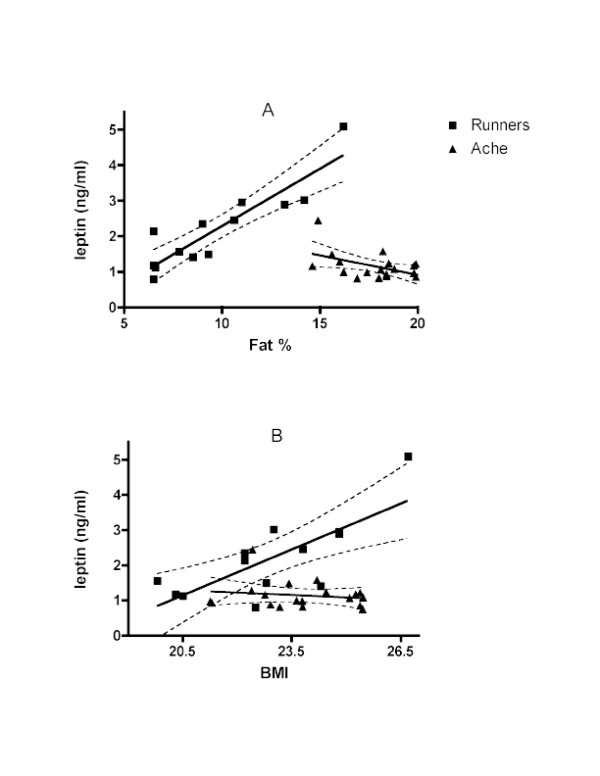
**Associations between leptin and anthropometric measurements**. Linear regression of leptin and fat % (a) in Ache men (r = -0.50, p < 0.03) and runners (r = 0.90, p < 0.0001). Slopes are significantly different (p < 0.0001, F = 40.0); (b) in Ache men (r = -0.17, p < 0.50) and runners (r = 0.75, p < 0.003). Slopes are significantly different (p < 0.001, F = 13.5).

Despite significantly higher body fat percentage (Ache 17.9 ± 1.78; Runners 9.7 ± 3.2; p < 0.0001), Ache leptin was significantly lower than runners (Ache 1.13 ± 0.38; runners 2.2 ± 1.15; p < 0.007). There was no significant difference in BMI (Ache 23.8 ± 1.4; Runners 22.9 ± 2.0; p < 0.16) or age (Ache 32.8 ± 16.1; runners 32.2 ± 9.2; p < 0.89) (Figures 2a–d). In addition, the associations between leptin and fat percentage were quite different. While runners exhibited the expected positive relationship between leptin and fat % (r = 0.90, p < 0.0001), the association in Ache men was negative (r = -0.50, p < 0.03), although the association is not significant with the elimination of one outlier (Figure 3a). Not surprisingly, the slopes were significantly different (p < 0.0001, F = 40.0). Similarly, leptin associations with BMI were highly significant among the runners (r = 0.75, p < 0.003) but not Ache (r = -0.17, p < 0.50). Again, the slopes are significantly different (p < 0.001, F = 13.5) (Figure 3b).

## Discussion

The hypothesis that leptin should be higher in the group exhibiting greater adiposity was not supported in this investigation. In addition, the hypothesis that leptin should be positively associated with adiposity is only evident among American runners. Therefore other sources of leptin variation, particularly among the Ache, need to be considered. The exact mechanisms that underlie population variation independent of adiposity remain unclear, however genetic differences between populations, chronic environmental influences such as maternal/fetal or childhood nutritional status, and acute lifestyle differences such as diet and physical activity may be contributory. Since American runners were chosen due to their leanness and physical activity compared to the Ache, it is unlikely that acute differences in daily lifestyle patterns related to adiposity and physical activity were a factor in this study.

Genetic differences in the Ob gene that codes for leptin as well as its receptor are also possible sources of variation. For example among Taiwanese aboriginal populations, greater incidences of obesity were associated with the G-2548A polymorphism in the promoter region of the leptin gene and the Gln223Arg (Q223R) polymorphism of the leptin receptor gene [18]. Similar findings are evident among Brazilian populations [19]. Variation in the proopiomelanocortin gene has also been associated with variation in leptin levels [20]. While various genetic analyses have been conducted among the Ache, descriptions of potential genetic leptin polymorphisms remain to be conducted. The Ache are a genetically homogeneous population and unique leptin polymorphisms may be possible [21].

McMillen and colleagues have emphasized the potential role of early developmental processes that may affect leptin and adipose physiology later in adulthood. They suggest high maternal BMI may lead to greater circulating glucose in fetuses and altered adipocyte metabolism during childhood, ultimately leading to greater leptin levels in adulthood and a propensity towards obesity [10]. However the inverse may be at play with the Ache. Among the Ache, maternal undernutrition may lead to lower adult leptin levels. One Ache woman, nine months pregnant, exhibited very low leptin levels (bottom 5^th ^percentile compared to American pregnant women) (unpublished data). However clearly further data on pregnancy and leptin are needed among the Ache.

In addition, a comparison of leptin between well-fed Italian and undernourished Gambian boys and girls revealed that Gambian children exhibited consistently lower leptin even after controlling for BMI [22]. Animal models have also shown that early exposure to leptin increases receptor formation in the hypothalamus [23]. Leptin clearance rates are also positively associated with adiposity, although relationships with population variation are unknown [24]. Conversely, a comparison of leptin among Pima Indian populations living under differing lifestyle and environmental conditions revealed that under conditions of high caloric intake/low energy expenditure (American) compared to low caloric intake/high energy expenditure (Mexican), leptin was significantly higher among the Mexican Pima independent of sex, adiposity, and insulin resistance state [25].

In adults, leptin levels and adipose tissue production rates exhibited a more prominent decline in fasted lean women compared to fasted obese women [26], while five days of intense active military training also decreased leptin levels in healthy men, most likely due to hypoinsulinemia and increases in catecholamines [3]. However both Ache and American runner men are very physically active. Therefore daily physical exertion may contribute to their low leptin levels compared to more sedentary men, but it does not account for the lack of association between leptin and adiposity among the Ache. Interestingly, elite male and female gymnasts exhibit hypoleptinemia and associated delays in puberty and other developmental challenges, suggesting continued effects on lower leptin production in adulthood [27]. However leptin responses to heavy endurance training exhibited only a modest increase in elite runners [28]. The Ache also undergo later puberty compared to American counterparts [29], intimating the potential role of energetic status during pubertal development and later adipose leptin production rates. Finally, zinc deficiency induces acute hypoleptinemia [30-33], however oral zinc supplementation did not affect Ache male leptin levels [34].

Immunological stress, while not quantified in this study, likely differed between Ache and American runner men. Chronic and acute infections affect and are influenced by leptin levels [5,35]. Specific aspects of Ache disease epidemiology are not well described. However, tuberculosis is becoming more common within the Ache community and may be a factor to consider [36]. Curiously, Yüksel and colleagues [37] reported that serum leptin levels were *higher *among active tuberculosis patients compared to controls. Treatment elevated leptin levels even higher. Schwenk et al. [38] also reported that wasting associated with tuberculosis was unrelated to leptin. It is therefore unlikely that possible tuberculosis infection made any significant contribution to Ache leptin profiles although the role of other infectious agents remain to be investigated.

While leptin likely serves as a fat cell mass/energy signal to the central nervous system, acute underfeeding can dramatically lower systemic leptin independent of changes in adipose tissue mass [2]. This is in accordance with the hypothesis that leptin may serve as an anti-starvation as opposed to an anti-obesity signal. The unusually low leptin in Ache males and females may be part of a coordinated metabolic milieu that is geared toward restrained energy intake and storage, as the environment is much more tenuous than a Western environment with abundant energy dense foods and little physical activity. Such a state is consistent with the thrifty genotype hypothesis and may contribute to contemporary high rates of obesity among Native American populations [39,40].

Foraging populations are excellent models for assessing the evolutionary implications of exercise physiology. Assessments of physical performance of the Ache have provided evolutionary physiologists with important insights into human biology and their responses to physical activity, both recreational and non-recreational [14]. Indeed, long distance running is a popular exercise activity may be rooted in the evolution of human physiology. Not only is skeletomuscular adaptation required, an efficient suite of metabolic mechanisms are also necessary to manage energy usage during this period of acute activity. Indeed, running has been suggested to have been a central factor in the evolution of habitual bipedalism [41]. Although investigations of our hominid ancestors are limited to examinations of the fossil record, research among contemporary hunter/gatherers and other non-industrial populations are useful models of the ecological, energetic, and lifestyle challenges that were probably not unlike those that confronted human ancestors. The effects of acute activity among forager populations suggests that chronic differences in lifestyle and ecological circumstance may influence physiological function [14,42].

The significant non-pathological range of variability in leptin and other metabolic hormones merits greater awareness and appreciation on the part of human biologists. Moreover, the growing incidence of obesity, metabolic syndrome, and diabetes, along with increasing population heterogeneity due to immigration, strongly advocates that diagnostic and research clinicians would surely benefit from recognizing the importance of leptin variation independent of adiposity.

## Conclusion

While adiposity has been repeatedly shown to be central to leptin production and circulating levels, we have provided further evidence that leptin levels exhibit a significant range of variation independent of somatic condition, most importantly adiposity. Leptin production rate varies in association with environmental conditions and contributes to population variation. Further research is necessary to ascertain the potential implications for differences in the incidence and etiology of obesity, diabetes, and other metabolic disorders among ethnicities and populations.

## Competing interests

The authors(s) declare that they have no competing interests.

## Authors' contributions

Dr. Richard G. Bribiescas initiated the research question addressed in this manuscript, performed the field collection and conducted the laboratory analysis of the Ache hormonal and anthropmetric data, performed the statistical analysis, as well as contributed to the preparation and editing of the manuscript. He also initiated and procured the grants necessary to collect the Ache data.

Dr. Matthew S. Hickey contributed to the collection and laboratory analysis of the American runner hormonal and anthropometric data. He also assisted in the preparation and editing of the manuscript. Both authors were involved with the preparation and editing of the final manuscript.
